# **Doctor Retention in Ireland - What It May Mean for the Global Health Workforce Reform Agenda** Comment on "Doctor Retention: A Cross-sectional Study of How Ireland Has Been Losing the Battle"

**DOI:** 10.34172/ijhpm.2020.126

**Published:** 2020-07-15

**Authors:** Bernard Hope Taderera

**Affiliations:** Department of Environmental Health, Faculty of Health Sciences, University of Johannesburg, Johannesburg, South Africa.

**Keywords:** Health Personnel, Policy, Retention, Migration, Ireland

## Abstract

The study of healthcare personnel migration in Ireland reports that most medical graduates plan to leave the country’s health system. It may be possible to address this challenge by understanding and addressing the reasons why young doctors plan to leave. Future studies should contribute to the retention of early career doctors in highincome countries such as Ireland. This will help reduce the migration of doctors from low- and middle-income countries in order to address the global health workforce crisis and its impact on the attainment of universal health coverage in all health systems.

The article on healthcare personnel migration in Ireland by Brugha, Clarke, Hendrick, and Sweeney contributes to an increasingly important discussion about the retention and migration of early-career medical graduates from some high-income countries. This paper also helps to understand what this may mean for the health workforce situation in low and middle-income parts of the world and the ongoing Human Resources for Health (HRH) reform policy efforts to address the global health workforce crisis. The study on Ireland reports the outbound migration plans of junior doctors in Ireland, why they leave and likelihood of them returning.^1^ This research not only helps understand the healthcare personnel challenges in Ireland but also provides an alternative viewpoint through which to understand how the prevailing global healthcare worker crisis is unfolding, and implications that this has for healthcare worker motivation and retention in healthcare human resources systems in developed, mostly the Organisation for Economic Co-operation and Development (OECD), and developing countries, mainly in sub-Saharan Africa and South East Asia.^2-6^ This article is also an important addition to the discussion about healthcare worker migration and what the failure to retain medical graduates in some first-world countries such as Ireland may mean in the endeavour to uphold principles set by the World Health Organization (WHO) Global Code on International Recruitment of Health Personnel.^7,8^ Further, and most importantly, this article contributes to the understanding of what the challenge of healthcare graduates’ migration may mean to the formulation, implementation and evaluation of retention strategies towards the realisation of HRH policy outcomes outlined by the 2018 Astana Declaration, and the HRH Global Strategy (Workforce 2030). This will have a positive impact in ongoing effort to attain the global healthcare worker reform policy goal of universal health coverage set by the WHO.^9,10^

My first comment is that it is a challenge for health personnel reform policy that most (more than half) of recently graduated young doctors in Ireland plan to leave the country’s healthcare delivery system, mainly for other high-income Anglophone countries.^1^ Perhaps it may be of increasing concern that a similar migration pattern has also been reported by other recent studies of Ireland, suggesting an immediate threat to the sustainability of the Irish healthcare delivery system.^2,3^ Most medical personnel who were reported to be planning to migrate, mainly to other high-income Anglophone countries, are emerging very young medical professionals. This means that there might be an inevitable prospect that Ireland will remain amongst the top 3 high-income countries relying on international recruitment of doctors from low-income countries in order to replace them.^1^ As a result, the HRH problems of Ireland may contribute towards continued doctor emigration from resource limited parts of the world. It is important for me to note that the migration of medical specialist graduates from low- and middle-income countries is not solely caused by Ireland. Rather globalisation, less favourable working conditions in the least developed parts of the world, health worker shortages in the face of increased demand for healthcare specialists mainly as a result of more favourable socio-demographic outcomes amongst the population in the more developed parts of the world and the portable nature of medical qualifications makes the migration of early-career medical doctors inevitable.^1-5^ My point in this is that healthcare personnel shortages and the failure to retain young medical doctors in any one country in the developed world is one driver of doctor migration from the already fragile healthcare worker systems in the third world. This pattern of health personnel migration has a less favourable effect on the global health personnel strengthening agenda, and indeed the pursuit of universal health coverage at a world level.

The voluntary nature of the Global Code of Practice on the International Recruitment of Health Personnel (the Code) might not make this situation better. For instance, domestic challenges in the Irish health personnel retention system prompting the migration intentions of medical graduates may undermine conformity to, in particular, Guiding Principle 3.6 under Article 3, prescribing members states to, as much as possible, create a sustainable health workforce and work towards retention strategies that will reduce their need to recruit migrant health personnel, and that if international recruitment of health personnel should be done, it be carried out in a manner that promotes the sustainability of health systems in developing countries.^7,8,11^ Further, retention challenges in Ireland may make it difficult to adhere to the Astana Declaration which asserts that the international migration of health personnel should not undermine countries’, particularly developing countries,’ ability to meet the health needs of their populations.^9^

My second comment is that in order to progress towards addressing this challenge, it is important to understand why young medical graduates plan to leave Ireland so as to stop them from leaving. The article by Brugha et al reports that the highest negative ratings among those planning to leave were for stress levels, staffing levels, training costs, protected training time, mentoring, non-core tasks, and bullying which was said to had become worse.^1^ Training expenses, limited training time, and stress are challenges that have also been reported mostly in resource constrained healthcare personnel systems in the developing world and they mainly emanate from critical healthcare worker shortages which make it very difficult for any health staff member to leave the duty station at health facilities to attend training at their convenience. This is mainly a result financial incapacity which makes it difficult for health systems in these low-income parts of the world to recruit, adequately remunerate and sustain the required number of skilled medical workers.^4-6^ Health financial challenges for Ireland might not be at the same level as those of the developing world but the health personnel retention and migration challenges appear to be similar. The migration plans of emerging medical doctors in Ireland may mean that there is a possibility that the country may fail to ensure the availability of adequately trained and motivated medical specialist personnel. This situation may undermine the delivery of quality healthcare services to the Irish society, a health workforce policy outcome set by the 2030 Global Health Workforce Strategy.^10^ The motivation of young medical graduates is important to ensure their retention in any healthcare system. For Ireland, like other high and low-income countries facing a similar challenge of outward migration by junior doctors, it may be necessary to get to the core of the triggers and drivers of this challenge so as to understand, commit adequate resources and address them.

With political will, system and structural organisation and financial resources, it may already be more feasible for a high-income country like Ireland to address stress levels, staffing levels, training costs, protected training time, mentoring, non-core tasks, and the bullying reported from the study by Brugha et al amongst its medical graduates. This might also be an effective and sustainable response to Ireland’s medical workforce crisis.^1^ Low-income countries, in contrast, might have the political will and institutional capacity (in terms of health personnel policies, structures and systems) but not the adequate financial resources to adequately address the emigration of skilled medical doctors and junior medical graduates.

My final comment is that there is need for health systems in high-income countries like Ireland to reinforce effort towards training and retaining adequate numbers of medical graduates. This will contribute towards stopping health personnel migration from low and middle-income parts of the world. However, prospects of that happening appear impossible because recent studies report that international migration of health personnel to high-income, mainly OECD and in more recent times some Asian countries is on the rise. Recent data reported from a study by Campbell et al suggests that there is an increasing reliance on foreign-born and foreign-trained health professionals in high-income countries with a 60% increase in the total number of migrant nurses and physicians over the last decade and a higher proportionate increase from countries experiencing critical health workforce shortages. These trends are projected to worsen as we progress into the future, towards 2030.^8^ Brugha et al in this article reported that there will be an estimated shortfall of 750000 doctors in 31 OECD countries by 2030, excluding an estimated shortage of 2.6 million doctors in poorer countries, from which countries like Ireland recruit their doctors. In addition, global health pandemics such as coronavirus disease 2019 (COVID-19) widen healthcare worker shortage gaps in all countries. To help address this situation, young Irish doctors are reported to have started to return home in their hundreds in March 2020 to support the COVID-19 response. Following this development, I agree with questions raised by Brugha et al that once the epidemic is under control, will Ireland take the steps to keep the young doctors who returned?^1^ Future research on Doctor retention in Ireland may also explore the following questions:

- How will health worker retention strategies by Ireland’s health authorities impact young Irish doctors post COVID-19;
- Will Ireland be successful to keep its very young medical graduates, and will this help mitigate Doctor migration from developing countries;
- What will be the effectiveness of Ireland’s health workforce strategies to address Doctor shortages after COVID-19; and
- What will the Doctor retention battle in Ireland mean for progression towards attaining health worker policy outcomes set by the 2030 Global Strategy on HRH?^7-10^

This study also provides some important lessons for the global health workforce reform agenda, in the endeavour to address the healthcare worker crisis, particularly in low and middle-income countries.^10^ The migration and shortage of medical doctors in developing countries has been reported particularly in Sub-Saharan Africa and South-east Asia.^3,5^ The study by Brugha et al reports that shortages and migration of young medical graduates in Ireland is caused by factors which include occupational stress, staffing levels, training costs, protected training time, mentoring, non-core tasks, and the bullying.^1^ These factors, in addition to unsatisfactory remuneration reported in low and middle-income countries, have long graduated from the systemic to the institutional global health workforce reform agenda of the world health system. The study of Ireland suggests that health systems across the world share similar health workforce challenges, particularly in relation to doctor migration. The WHO has formulated the 2030 Global Health Workforce Strategy, from which declarations are made based on periodical review.^9,10^ There remains a need, from this global health workforce agenda, to reinforce the formulation and implementation of health worker population category, country and/or regional specific interventions to retain and mitigate the migration of medical graduates: (*i*) between high-income countries; and (*ii*) from low to middle and/or high-income nations. Funded training schemes and bonding are policy options for Ireland. Retention in the period after the bonding period is also equally important in order to help ensure the availability of healthcare workers to meet the increasingly personalised needs of a population with a higher life expectancy. Another strategy to reduce a heavy workload on doctors is by shifting non-core task responsibilities to other non-medical personnel where possible.^1,3^ This will help create space for senior specialists to provide mentoring to junior doctors, and provide time for further on the job training.

Another lesson is that the Irish study by Brugha et al provided four criteria of migration intention namely: (*i*) remain; (*ii*) go abroad and return; (*iii*) go and stay abroad; and (*iv*) quit the profession.^1^ These criteria are a very useful way through which future studies may help generate understanding of the migration intentions of physicians in low- and middle-income countries, where the implementation of retention strategies can be reinforced. The Irish study also made use of a low-cost online methodological approach through which data was collected. This data collection approach can also be used in low- and middle-income countries to assess medical graduates intentions to migrate. The approach is a very useful way through which to collect data particularly in situations where there are financial resource constraints or where human contact is limited by highly infectious pandemics, such as COVID-19. However, perhaps the weakness with this data collection approach is that it is not feasible in resource constrained areas where computers and internet connection are limited. In addition, the Irish study suggests that the response rate associated with this online data collection method might be low which may affect the credibility of the dataset as researchers may be forced to draw conclusions based on how many responses they are able to receive which might be less than the actual sample size. Then also there is no benefit of understanding the lived experience of participants which may be possible in face to face data collection means. Nevertheless, findings by Brugha et al reveal results similar to those reported in similar studies that have been carried out on Ireland. This helps understand the migration intentions of young medical graduates and implications that this has for doctor retention in Ireland.^1,2^

## Ethical issues

 Not applicable.

## Competing interests

 Author declares that he has no competing interests.

## Author’s contribution

 BHT is the single author of the paper.
